# Real-Time Sensor-Based and Self-Reported Emotional Perceptions of Urban Green-Blue Spaces: Exploring Gender Differences with FER and SAM

**DOI:** 10.3390/s25030748

**Published:** 2025-01-26

**Authors:** Xuan Zhang, Haoying Han, Guoqiang Shen

**Affiliations:** 1 College of Civil Engineering and Architecture, Zhejiang University, Hangzhou 310058, China; zhang_xuan@zju.edu.cn (X.Z.); gshen214@zju.edu.cn (G.S.); 2 Faculty of Innovation and Design, City University of Macau, Macau 999078, China; 3 Center for Balance Architecture, Zhejiang University, Hangzhou 310028, China

**Keywords:** environmental quality, gender difference, urban green-blue space, emotional perception, facial expression recognition, semantic segmentation, deep learning, Google street view, urban planning

## Abstract

Urban green-blue spaces (UGBS) are increasingly recognized for their benefits to physical and mental well-being. However, research on real-time gender-specific emotional responses to UGBS remains limited. To address this gap, a dual-method approach combining facial expression recognition (FER) and self-reported measures to investigate gender differences in real-time emotional evaluations of UGBS was developed. Using static images from Google Street View as stimuli, a self-reporting experiment involving 108 participants provided insights into subjective emotional experiences. Subsequently, a FER experiment, utilizing 360-degree video stimuli, captured over two million data points, validating the feasibility and advantages of real-time emotion monitoring. The findings revealed distinct gender-specific emotional patterns: women experienced stronger pleasant emotions and preferred scenes evoking higher arousal, while men demonstrated sharper responses and rated scenes with peak valence emotions more favorably. Grass elicited relaxation and delight in women and arousal in men, whereas blue spaces induced calmness across genders, with men reporting greater relaxation as water content increased. The study underscores the potential of FER technology in assessing real-time emotional responses, providing actionable insights for inclusive urban planning. By integrating advanced tools and participatory design approaches, urban planners can develop strategies that enhance emotional well-being and create livable cities that support diverse user needs.

## 1. Introduction

The COVID-19 pandemic led to the global implementation of measures such as physical distancing, mobility restrictions, and social isolation, resulting in profound impacts on mental health and significant disruptions to daily routines [1,2,3,4]. This period has also underscored the critical importance of physical and mental well-being. Numerous empirical studies have highlighted that urban green-blue spaces (UGBS), characterized by features such as vegetation, water bodies, and open skies, contribute positively to physical, psychological, and emotional health outcomes [5,6,7,8,9,10]. As UGBS could play a role in promoting the gender-related “right to the city” and health equity, there is a growing need for more rigorous empirical research to develop equitable design preferences and inclusive interventions in public spaces [11,12,13].

Previous research has identified gender variations in several aspects related to UGBS, including preferences, restoration and health outcomes, safety perception, housing demand, and usage patterns [12,14,15,16,17]. The observed gender distinctions in the physical and mental health benefits associated with exposure to green spaces may be due to gender-specific perceptions and uses of these urban environments [18,19]. An important indicator of popular approval of UGBS is the expression of aesthetic emotions, reflecting individual aesthetic judgments [20,21]. Research indicates that gender disparities exist in emotional responses, with females more susceptible to mood disturbances and showing enhanced emotion-specific physiological reactions [22,23,24,25], whereas males demonstrate superior cognitive management of negative emotions and employ cognitive control tactics to mitigate these effects [26,27,28].

In recent years, Street View Images (SVIs) have gained prominence as a tool for researchers to analyze visual perceptions of the built environment from a human-centric perspective over large-scale areas [29,30,31]. Among these, Google Street View (GSV) stands out as a widely used open-source platform, providing panoramic and detailed representations of urban streetscapes from a pedestrian viewpoint. Its application has been integral to numerous studies on urban perception [32,33,34,35]. Previous research, such as studies by Bradley et al., has extensively explored emotional gender differences in image perception using the International Affective Picture System (IAPS), revealing distinct responses between men and women, especially towards negative images [36,37].

Technological advancements have enabled sophisticated analyses of urban environments by integrating diverse big data sources and crowdsourced surveys [38,39]. To classify emotions, researchers commonly use two frameworks: the seven basic emotions (e.g., happiness, fear, and anger) and the two-dimensional model of valence and arousal, which assess pleasantness and emotional intensity, respectively [40]. While traditional methods such as the Self-Assessment Manikin (SAM) scale rely on self-reported data to evaluate valence and arousal [41], there is a growing emphasis on dynamic and real-time monitoring of emotional responses [42,43]. Physiological sensors, including skin conductance [44], EEG [45], and eye-tracking technologies [46,47], have been employed to assess emotional reactions to landscapes. Concurrently, advancements in machine learning and facial expression recognition (FER) technologies [35,48,49] have enriched the toolbox for studying emotional responses to landscapes. For instance, geotagged facial images from social media have been utilized to map spatial emotional distributions and infer urban emotional dynamics [50,51,52]. In controlled laboratory settings, FER enables precise and unobtrusive monitoring of emotional responses to specific stimuli, surpassing the limitations of electrode-based methods [48,53]. Together, these approaches deepen our understanding of human emotional perceptions, offering valuable insights for urban design and environmental psychology.

Despite advancements in evaluating human emotional perceptions towards UGBS, the majority of studies have predominantly relied on self-reported measures and physiological indicators, often overlooking the significance of real-time perception [41,42,48,52,54]. This omission is notable, as real-time perception provides a more dynamic understanding of how individuals interact with their environment. Furthermore, the role of gender in shaping these real-time perceptions of UGBS has been particularly underexplored, despite its critical relevance to urban livability and the creation of inclusive urban spaces [55,56]. Hence, this study aims to answer the research question: How do gender differences manifest in real-time perceptions of UGBS? To support this inquiry, we establish two sub-questions: How does FER compare with subjective self-report measures in capturing gender differences in real-time perceptions of UGBS? What additional insights can FER provide into the dynamics of gender-specific emotional responses that may be obscured by self-report measures alone?

To address these research gaps, our study employs a streamlined methodological approach, encompassing the creation of visual stimuli, extraction of key visual elements, collection of both self-reported and real-time facial emotional responses, and a comparative analysis to elucidate gender differences in the perception of UGBS.

This study contributes to the body of knowledge in four key aspects. First, it addresses the critical gap in understanding gender differences in real-time perception of UGBS by employing FER technology, capturing spontaneous emotional responses from both male and female participants as they interact with virtual UGBS environments. Second, the study provides a comparative analysis between real-time FER data and traditional self-reported measures, shedding light on their congruence and divergence in reflecting gender-specific reactions to UGBS, and highlighting FER’s ability to capture authentic emotional expressions. Third, it introduces an open-source data pipeline for street-view images, establishing a research framework that is readily transferable to various settings and countries. Fourth, the study contributes to the development of urban greening strategies aimed at fostering inclusive and human-centric urban environments.

The paper is structured as follows: Section 2 details the methods employed in the study, including the preparation of visual stimuli, the extraction of visual variables, the conduct of self-report and FER experiments, and the approach to data analysis and comparison. Section 3 presents the findings from the comparative analysis of FER data and self-reported measures, highlighting the gender-specific emotional responses to UGBS and the effectiveness of different methodologies. Section 4 interprets the findings and discusses their implications for the development of inclusive urban greening strategies. Section 5 concludes the paper.

## 2. Materials and Methods

### 2.1. Research Framework

**Figure 1:**
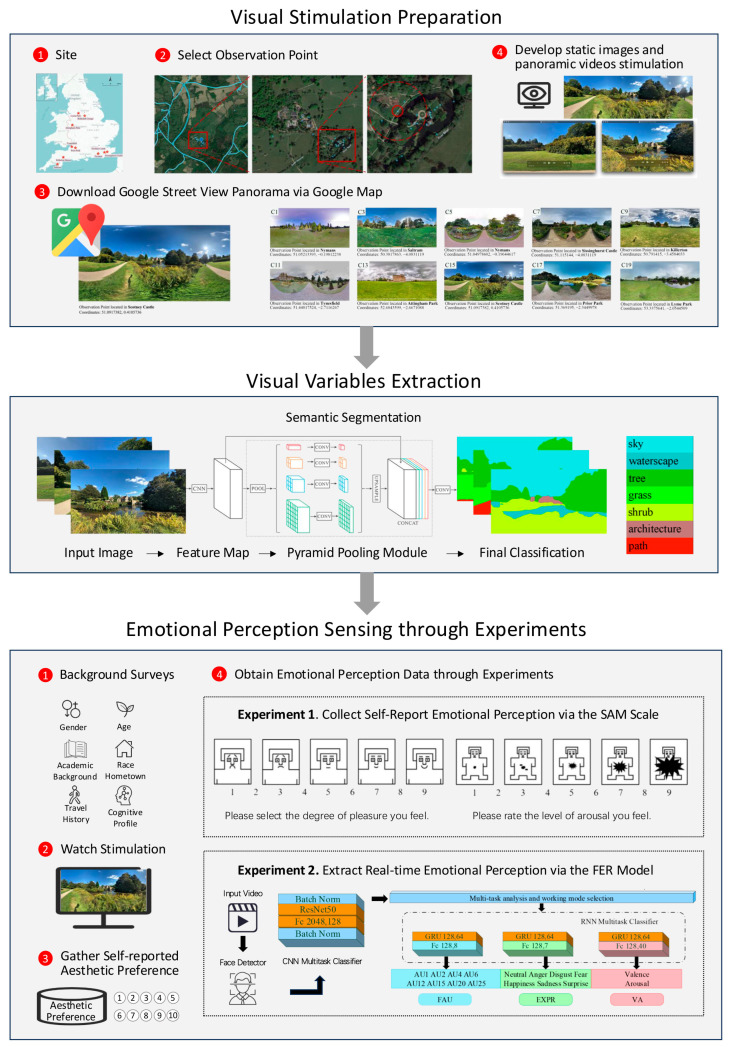
Research framework. For more details, refer to Questionnaires S1–S3 and Figures S1–S3.

Figure 1 outlines the structured methodology of our study, which is designed to explore gender disparities in the perception of green spaces. Our research is methodically divided into five distinct yet interconnected steps, ensuring a systematic approach to data collection and analysis:

1. Visual Stimulation Preparation: We employed the GSV platform (Google, CA, USA) to select and capture panoramic images at specific sites, which were then developed into both static images and panoramic videos. These visual stimuli were utilized in the subsequent self-report and facial expression experiments to elicit emotional responses.
2. Visual Variables Extraction: We utilized a scene semantic segmentation model to extract key visual variables of the captured videos and photos.
3. Emotional Perception Sensing through Experiments: A cohort of 108 participants was presented with the static images and asked to articulate their emotional responses using the validated SAM scale, providing a quantitative measure of their subjective emotional experiences. A separate cohort of 20 participants viewed the panoramic videos, and their facial expressions were captured and analyzed using a FER model, allowing for the objective assessment of their real-time emotional reactions.
4. Data Analysis and Comparative Evaluation: We conducted a comprehensive integration of the extracted visual variables with the data from facial emotional perception, comparing these insights with the self-reported emotional responses. This comparative analysis aimed to elucidate the impact of visual variables on gender-based perception disparities and to assess the relative effectiveness of different methodologies in capturing emotional responses to green spaces.

### 2.2. Visual Stimulation Preparation

To highlight the role of visual variables in emotional reactions to green spaces, British Heritage landscapes were selected as the main visual stimuli, ensuring that the elements of the landscape were distributed in a cohesive and uniform manner. These landscapes, characterized by impressive architecture, vast grasslands, and tranquil lakes, were ideal for maintaining a concentrated representation of landscape features. The primary stimuli were sourced from the National Heritage List for England (NHLE), an authoritative catalog of significant cultural and historical assets, including buildings, parks, gardens, monuments, shipwrecks, and World Heritage Sites within the UK.

To prevent emotional fatigue and sustain participant engagement during exposure to the main stimuli, contrasting auxiliary stimuli were introduced. Japanese landscapes, with their fragmented layouts and distinctive styles, were chosen to provide a striking difference from the primary British scenes. These auxiliary visuals were derived from Japan’s designated Special Places of Scenic Beauty, Special Historic Sites, and Special Natural Monuments, recognized under the Law for the Protection of Cultural Properties by the Minister of Education, Culture, Sports, Science, and Technology (MEXT). This approach ensured a balanced emotional experience and increased the diversity of visual exposure in the study [48].

To create a comprehensive and authentic experimental environment, we used both GSV images and panoramic video clips, ensuring consistency through a rigorous standardization process. A data-driven approach guided the site selection process, using specific garden names from the pre-determined lists of British and Japanese heritage landscapes as search terms on Instagram (Meta, CA, USA). By searching these garden names as hashtags (e.g., #lymepark, #scotneycastle), we ranked the results based on the number of posts associated with each hashtag (data collected on 5 April 2024). This approach allowed us to identify popular gardens with significant public interest [57,58]. From the top-ranked locations, we selected target gardens and identified optimal observation points within each site for acquiring high-quality street view imagery. Some scenes were excluded due to factors such as inadequate lighting, poor weather conditions, or suboptimal image quality. The final set of visual stimuli represented the best combination of popularity and visual clarity.

Utilizing the Street View Download 360 Pro (version 3.1.3) and the GSV API, we extracted high-quality, visually representative panoramas from these selected observation points (Figure 1). All video clips were processed with the same parameters: resolution 1920 × 1080, field of view 70°, pitch 0°, frame rate 30 FPS, duration 24 s, with clockwise spin for primary stimulation and anticlockwise for auxiliary stimulation. In line with experimental protocols that require varied exposure to scenes, the panoramic clips were generated and assembled into the final stimulation form in a flexible manner, allowing for randomization of scene presentation to participants.

### 2.3. Visual Variables Extraction

This study concentrates on eight visual variables that are closely associated with the perception of green spaces. These include the Green View Index (GVI), Visible Plant Index (VPI), and the relative proportions of trees, grass, shrubs, water bodies, sky, and buildings. We conducted pixel-wise semantic segmentation on each panorama and frame of the primary video stimuli using a Pyramid Scene Parsing Network (PSPNet)-based model, which leverages a ResNet-101 backbone for deep supervision. This approach is well-documented in the literature for its effectiveness in pixel-level feature extraction [59,60].

The PSPNet model, trained on the ADE20K dataset that encompasses 150 categories and 1038 scene descriptors, was fine-tuned to recognize seven specific classes relevant to our experimental setup, as depicted in Figure 1 [59,61,62,63]. The ADE20K dataset is a comprehensive resource for scene parsing tasks, providing a robust foundation for training deep learning models to accurately segment and classify visual elements within complex scenes [62].

Our methodology for extracting visual variables ensures that the features captured are representative of the landscape elements that contribute to the perception of green spaces. This rigorous extraction process is pivotal for subsequent analyses, which aim to correlate visual variables with emotional responses and aesthetic preferences.

### 2.4. Emotional Perception Sensing Through Experiments

#### 2.4.1. Emotional Self-Report Experiment via the SAM Scale

We recruited 108 participants (52 males and 56 females) for the emotional self-report experiment, with ages ranging from 18 to 43 years and an average age of 31.0 years (standard deviation = 8.2 years) (Table 1).

Prior to the visual stimulation experiment, participants completed a comprehensive survey to collect demographic data and individual experiences. This information is crucial for identifying potential correlations between participants’ backgrounds and their subsequent reactions to the visual stimuli.

Subsequent to the background questionnaire, participants were presented with a series of panoramic images and were asked to rate their emotional responses using the SAM scale and their aesthetic preferences on a scale from one to ten for each scene. This methodology enabled a nuanced assessment of the participants’ emotional and aesthetic reactions to the visual stimuli.

#### 2.4.2. Real-Time Emotional Recognition Experiment via Facial Expression Analysis

This experiment involved 20 participants, evenly distributed across genders, with no formal education in systematic planning or tourism and no prior visits to the UK or Japan. Participants, aged 20 to 26 with an average age of 23.4 years and a standard deviation of 1.5 years, were selected to represent a homogeneous group for initial exploratory studies, a common practice in FER research due to the resource-intensive nature of such studies [48,64,65] (Table 1). A pre-trained multi-task CNN-RNN architecture was employed to estimate valence–arousal dimensions, which were tailored to our research objectives. The model, initially fine-tuned with an Adam optimizer at a learning rate of 0.0001, was adjusted by a factor of 10 every three epochs.

The facial expression analysis process, depicted in Figure 1, commenced with the importation of video source data into the model. A face detector identified facial expressions frame-by-frame, which were subsequently sent to the CNN multitask classifier for feature extraction and categorization. The extracted features were then directed to the task working mode selector, which allocated them to one of the three distinct RNN components for specific tasks (FAU, EXPR, and VA), ultimately delivering the final results through a fully linked layer [51,52,54,66].

**Figure 2:**
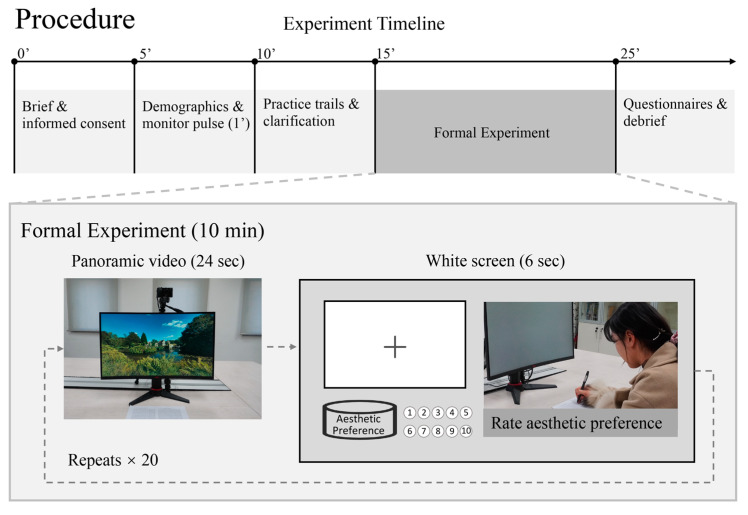
Experimental Procedure.

The experiment was conducted under controlled laboratory conditions, following the standardized procedure outlined in Figure 2. The laboratory was carefully arranged to provide a comfortable and quiet environment, helping participants feel at ease. To minimize the potential impact of environmental variations on FER accuracy, the room temperature was controlled within a range of 22 °C to 25 °C, and the lighting conditions were maintained uniformly throughout all sessions [65].

Upon their arrival in the laboratory, participants were seated in front of a monitor at eye level and briefed on the experimental procedures. A camera was positioned to capture their facial expressions throughout the entire experiment. Following a background questionnaire, participants were asked to relax and monitor their pulse for one minute to acclimate to the experimental environment.

The experiment then commenced, with primary stimulation presented in a randomized order to ensure an equal distribution of stimuli among participants, as shown in Figure 1. During the presentation of each panoramic video, participants were instructed to maintain their gaze on the screen, enabling consistent and uninterrupted facial expression data collection via the camera. Between video clips, a white screen was displayed as a transition, during which participants were asked to rate their aesthetic preferences of the scene they had just viewed on a scale from one to ten (Figure 2). This structured interval ensured that the self-reported ratings did not interfere with the real-time facial expression analysis, as the evaluation occurred after the stimuli were displayed and recorded. The combination of these measures ensured the independence and reliability of the facial expression data and the aesthetic preference ratings.

### 2.5. Data Analysis and Comparison

We initiated our analysis with a descriptive comparison of emotional and aesthetic responses across video clips for both genders, utilizing three perception measures. Outliers in the dataset were carefully identified and addressed to ensure the reliability of the analysis. Specifically, outliers were defined as data points exceeding 3 standard deviations from the mean or falling beyond 1.5 times the interquartile range (IQR). Boxplots and residual analyses were used to detect outliers in valence, arousal, and aesthetic preference ratings. Data errors caused by technical issues, such as FER misclassification, were excluded, while genuine extreme responses were retained but analyzed separately to assess their influence on the results. Sensitivity analyses confirmed that the exclusion of these points did not affect the main trends or conclusions. Given that video clip C3 produced illogical responses due to intermittent pauses, its data were excluded to mitigate potential bias.

This was followed by an analysis of real-time emotional perception trends and extreme peaks to assess the influence of urban characteristics on gender-specific FER responses. Pearson correlations were calculated to explore the relationships between dominant landscape features and participants’ perceptions.

Furthermore, we conducted a backward multiple linear regression analysis, considering three sets of visual variables with increasing granularity as independent variables and the two-dimensional FER perception data as dependent variables. The study also examined the correlation between aesthetic preferences and the two emotion dimensions by integrating demographic data, individual experiences, and cognitive profiles, thereby deepening the analysis.

The inclusion of 108 participants’ self-assessed emotions served as a crucial supplement to the FER data. This dual-method approach allowed us to compare immediate, unconscious emotional responses with conscious, reflective assessments provided by the participants. The comprehensive and comparative analysis not only broadened our understanding of participants’ perceptions but also evaluated the effectiveness of the FER-aided method in comparison to traditional self-reporting techniques.

## 3. Results

### 3.1. Gender Differences in Perception Measures

Significant gender differences were observed in perception metrics across nine scenes, highlighting marked emotional and aesthetic responses to UGBS. Table 2 compares these differences, using data from self-reported emotions and facial expressions. The findings align with our research aim to explore how these differences manifest in real-time perception.

**Figure 3:**
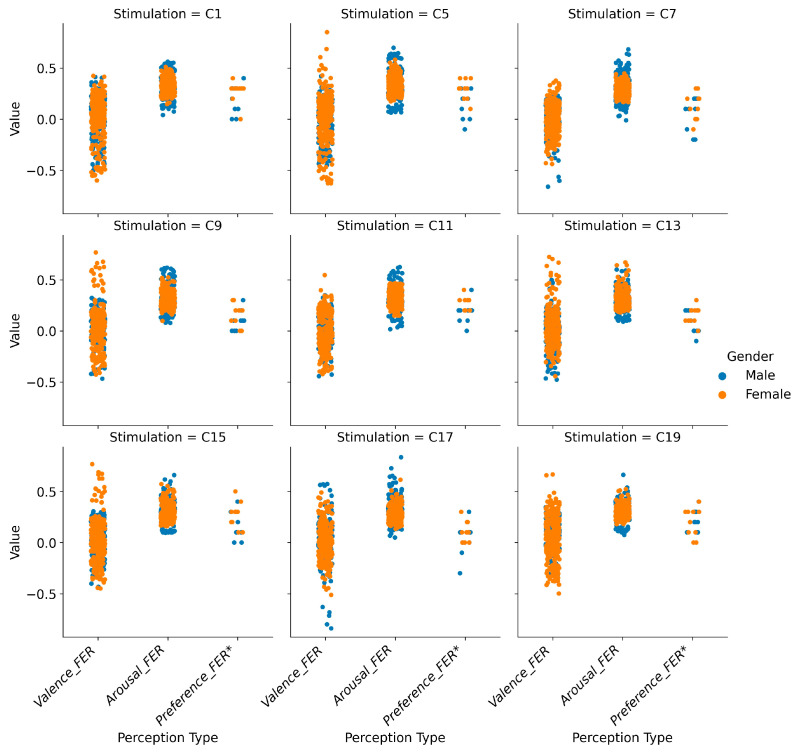
Gender differences in the FER perception measures for the nine clips were analyzed. For a valid comparison, the Preference_FER scores were converted into Preference_FER* scores, standardized to the same range as Valence_FER and Arousal_FER (−1.00 to 1.00).

Emotional responses to UGBS varied considerably between genders, particularly in valence scores, as illustrated in Figure 3. Arousal scores showed a predominantly positive and concentrated pattern across all videos, while valence scores exhibited significant variability, reflecting the complex interplay between environmental stimuli and emotional responses.

The FER approach revealed deeper insights into gender-specific differences than the SAM scale and overall scene preference ratings [44]. Women’s valence scores displayed greater variability, with peaks in scenes C5, C9, and C15. In contrast, men showed significant lows in scenes C7 and C17, as well as high arousal scores for scene C17, suggesting a strong emotional reaction to specific environmental features.

These findings emphasize the distinct ways in which men and women emotionally perceive UGBS. Section 3.2 will further dissect these gender-specific patterns to explore the factors driving emotional perception peaks.

### 3.2. Gender Differences in Real-Time Emotional Trends and Peaks

**Figure 4:**
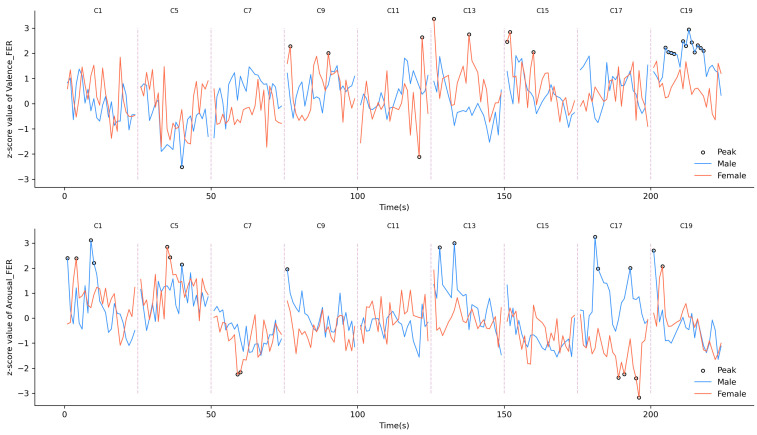
Real-time FER emotional perception and peak data for both genders.

Real-time emotional responses reveal pronounced gender differences in valence and arousal trends, offering critical insights into emotional perception dynamics. Figure 4 presents the moment-to-moment FER emotional z-score results, highlighting peaks (z-scores > 1.96) and providing a comprehensive overview of gender-specific emotional trends in response to primary stimuli.

Although men and women displayed broadly similar emotional trends, significant differences emerged in their reactions to specific video clips. Men exhibited thirteen valence peaks, primarily during scene C19, while women recorded nine. In arousal, men reported eleven peaks compared to nine for women, underscoring subtle but important gender-specific variations.

Analysis of specific scenes revealed that certain environmental features elicited different emotional responses between genders. Spacious grass areas, such as those in scenes C1, C9, and C13, led to arousal peaks in men while evoking valence peaks in women. Similarly, attractive buildings in scenes C9 and C13 produced positive valence peaks but negative arousal responses in women, indicating a complex interaction between aesthetic features and emotional intensity. Flowers, as seen in scene C5, triggered arousal peaks in both genders, but men’s negative valence scores suggest a mixed emotional experience.

Water-related features also highlighted evident gender differences. In scene C15, the initial presence of grass dominated the visual frame and led to valence peaks in women. However, as water content increased, both valence and arousal responses decreased for women. Conversely, in scene C19, the rising water proportion correlated with twelve valence peaks in men, suggesting that water features evoke stronger positive emotional responses in men under certain conditions.

These findings highlight the interplay between arousal, valence, and specific environmental elements, proposing hypothetical relationships that warrant further exploration [22,23,24,25]. Future studies could explore how gender differences influence emotional perception of environmental features to draw more definitive conclusions.

### 3.3. Gender Differences in Correlations Between Emotional Perception and Visual Variables

#### 3.3.1. Gender Differences in Pearson Correlations

**Figure 5:**
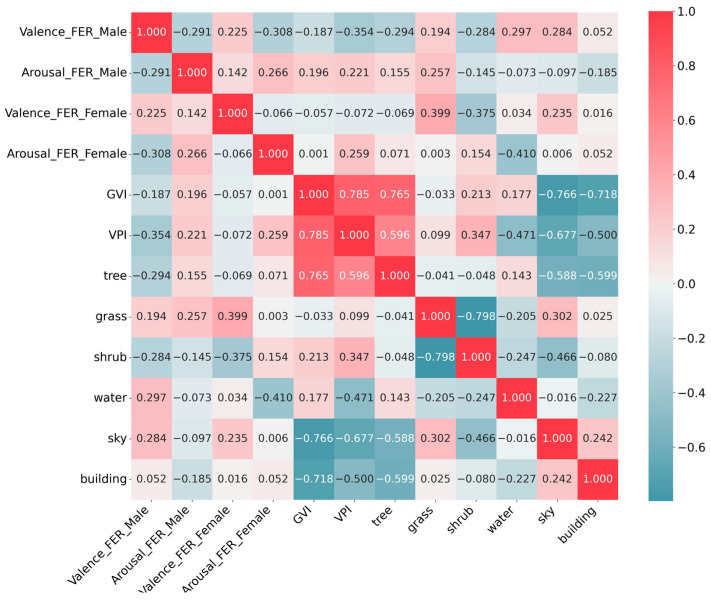
Heatmap of Pearson correlations between visual variables and FER emotional perceptions.

Our analysis of the correlations and regressions between visual variables and perception data revealed significant gender differences in how urban features impact perception, which is a key aspect of understanding gender-specific responses to UGBS. Contrary to initial expectations, we found no significant links between self-reported SAM emotions, preferences, and the visual variables extracted from panoramic images. This finding suggests that self-reported measures may not fully capture the nuances of emotional responses to environmental stimuli. However, the FER analysis provided insights into valence and arousal that may elucidate the underlying motivations behind individuals’ evaluations. The Pearson correlation coefficient, computed for all primary stimulation, showed gender-specific variations in the correlations between visual variables and the two dimensions of FER emotional responses, as visualized in Figure 5.

#### 3.3.2. Gender Differences in FER Perception Prediction Models with Three Different Combinations

Our research utilized backward multiple linear regression analysis to delve into the correlations between visual variables and FER emotional perception, employing three sets of visual variables with increasing granularity as independent variables (Table 3, Table 4 and Table 5). This approach allowed us to uncover distinct gender differences in how these variables predict emotional responses, a key focus of our study.

Model 1, which included GVI, Sky, and Building as independent variables, indicated that these variables accounted for approximately 7.6% and 13.5% of the variation in valence perception data for men and women, respectively (Table 3). This finding suggests that while a modest proportion of the variance in emotional responses can be explained by these variables, there is a significant gender disparity in how they influence valence perception.

Model 2, which separated VPI and Water from GVI and included them as independent variables alongside Sky and Building, led to a significant increase in the adjusted R^2^ for arousal data in both genders, from 0.000 to 0.178 for females and from 0.034 to 0.045 for males (Table 4). This enhancement in model fit highlights the importance of these variables in predicting emotional arousal, particularly for females. Water and Sky emerged as the strongest predictors of valence for both genders, with Building also positively influencing female valence outcomes. VPI was identified as a key indicator for arousal across genders, with significant associations observed between sky and building variables and high levels of female arousal (*t* = 4.541 ** and *t* = 3.688 **, respectively).

In Model 3, after further dividing VPI into Tree, Grass, and Shrub, six detailed variables were employed as independent variables, resulting in the greatest explanatory power compared to the previous models (Table 5). The adjusted R^2^ for valence in males notably increased to 0.258, indicating that these variables accounted for 25.8% of the variation in the valence dimension for men. For females, these variables explained 17.5% of the variation in the valence dimension. Additionally, the adjusted R^2^ for arousal in males increased to 0.108, and for women, to 0.245. The gender-specific analyses revealed markedly divergent outcomes; for valence, the proportion of grass was the sole predictor for women’s outcomes, whereas for men, all visual variables except the proportion of water had negative associations with the outcomes. For arousal predictions, all variables exhibited negative impacts on males, while for women, these variables demonstrated positive effects. Notably, water content predominantly influenced men’s valence (*t* = 4.097 **), with a negative outcome for arousal (*t* = −3.073 **). For women, valence outcomes were primarily determined by the proportion of grass (*t* = 5.813 **), with positive results for arousal as well (*t* = 6.480 **).

These results underscore the complex and gender-specific relationships between visual variables and emotional perception, providing valuable insights into how different environmental elements may elicit distinct emotional responses based on gender.

### 3.4. Gender Differences in the Interrelationship Between Aesthetic Preference and Perception Data

#### 3.4.1. Gender Differences in the Interrelationship Between Aesthetic Preference and Self-Reported Perception Data

The relationship between aesthetic preference and self-reported perception data appears consistent across genders, with no significant gender-specific trends identified. Within both male and female groups, significant correlations were observed between self-reported valence, arousal, and preference ratings (Table 6). These findings suggest a close link between individuals’ emotional responses and their aesthetic evaluations of the environment.

However, when participants assess their experiences using the SAM scale, these ratings may reflect an overall perception of the scene rather than capturing subtle gender differences in emotional responses. The SAM scale, as a static and holistic measure, may not fully reveal the dynamic emotional variations between genders. In contrast, dynamic measures like FER can detect instantaneous emotional changes that self-reported measures may overlook, highlighting the value of complementary methodologies in uncovering significant gender disparities.

#### 3.4.2. Gender Differences in the Relationship Between Aesthetic Preference and FER Perception Data

Our analysis explored the correlations between aesthetic preference and the highest, average, and minimum FER emotional response values (Table 7), revealing significant gender-specific differences. These findings highlight how emotional response patterns influence aesthetic preferences differently for men and women.

For male participants, the highest valence score for each clip was positively and significantly correlated with aesthetic preference (*p* < 0.05), suggesting that peak moments of positive emotion strongly influence their aesthetic evaluations. In contrast, for female participants, the average arousal score for each clip showed a positive and significant correlation with aesthetic preference (*p* < 0.01), indicating that sustained emotional arousal is a key factor in their aesthetic judgments.

These findings underscore the different ways in which emotional responses and aesthetic preferences are linked, further highlighting the importance of accounting for gender-specific emotional dynamics when studying perceptions of UGBS.

## 4. Discussion

### 4.1. Comparative Analysis of FER and Self-Reported Measures in Capturing Gender-Specific Emotional Perceptions

Our study leveraged FER technology to detect gender differences in emotional reactions to urban visual elements, revealing significant distinctions based on gender. The FER analysis, which captured participants’ facial responses, provided comprehensive and dynamic emotional perception data at the frame level. This approach, employing valence and arousal metrics, offered a deeper understanding that surpasses traditional image-level or video-level assessments, thus providing more nuanced insights than aesthetic preference evaluations and SAM self-reported assessments [44].

The FER data highlighted that the lag between participants’ perceptions and their SAM reporting might result in aesthetic preferences that do not accurately reflect their true emotions. Our findings revealed no direct correlation between the visual elements of panoramic UGBS images and participants’ self-reported SAM emotions, suggesting that subjective evaluations may not fully capture the nuanced emotional responses triggered by visual stimuli.

By capturing real-time emotional changes at the frame level, FER allowed us to reliably assess unconscious response fluctuations and short-lived emotional changes, overcoming the limitations inherent in self-reported ratings. This divergence from previous studies that focused on cognitive and neural responses to image perception underscores the positive emotional impacts and therapeutic benefits of UGBS.

Interestingly, our study found that men’s aesthetic preferences were influenced by the highest valence scores of clips, while women’s preferences correlated positively with average arousal levels. This indicates distinct emotional processing and aesthetic appreciation between genders, with men potentially giving higher overall ratings to scenes that evoke the most pleasant emotions, and women assigning higher ratings to scenes that elicit strong arousal.

### 4.2. Gender-Specific Emotional Responses to UGBS Visual Variables

Utilizing deep learning, our research conducted an in-depth analysis by associating emotions with urban features on a frame-by-frame basis. Overall, both genders exhibited significant perception changes in response to alterations in visual variables, with notable gender differences: women generally experienced more intense positive emotions, while men showed quicker arousal responses to changing visuals. This approach successfully pinpointed moments of emotional transition, accurately identifying instances where participants’ emotions exhibited subtle changes, thereby detecting subtle visual cues.

Grassy areas were found to evoke intense pleasantness in women and rapidly arouse men’s emotions, indicating distinct emotional responses to green space elements across genders. These results align with previous research highlighting a substantial correlation between the visibility of grass in an image and its stress-reducing, mentally restorative, and positive emotional response-inducing capabilities [67,68].

Waterscapes, known for their healing and wellness benefits, play a crucial role in therapeutic landscape design [9,69]. Research on their impact on well-being, especially considering gender differences, remains limited. Our study finds that blue spaces may induce calmness across genders but with notable differences. With an increase in water content, women may experience decreases in both valence and arousal levels, suggesting an intensified feeling of calmness. In contrast, for men, a higher proportion of water correlates with increased pleasure and reduced arousal, signaling a more profound relaxation.

Scene configurations can evoke diverse emotional responses. The style of building may influence the emotional responses of participants, with noticeable variations among women. Well-designed flowerbeds impact emotions differently, inducing significant mood shifts in both genders. Not all natural elements evoke universally positive reactions; for instance, vivid flowers may trigger negative responses due to perceived visual clutter, indicating that the organization and complexity of a scene are critical in determining its emotional impact. These findings underscore the importance of considering design configurations’ emotional effects in future research [70].

These gender-specific responses to UGBS visual variables lay the groundwork for crafting urban design strategies that are more nuanced and attuned to the emotional well-being of all users. By considering these differences, urban design can become an instrument for enhancing emotional well-being, leading to the creation of more inclusive and livable cities that cater to the diverse emotional landscapes of their inhabitants.

### 4.3. Implications for Future Urban Planning and Inclusive Urban Design

The gender disparities in emotional responses to UGBS revealed by our study underscore the importance of considering emotional well-being in urban design. Urban designers must prioritize the emotional needs of all users, particularly in spaces intended to foster health and well-being. For example, in settings such as parks, hospitals, or community healing gardens, increasing grassy areas can enhance pleasant experiences for women and foster rapid emotional engagement among men. Strategically incorporating green elements, like open lawns and tree canopies, can maximize their restorative potential while maintaining visual harmony.

Targeted planning of blue spaces is also essential in contexts demanding tranquility and relaxation, such as waterfront promenades or wellness centers. The inclusion of diverse environmental features, such as water bodies, greenery, and open spaces, should be carefully tailored to cater to the distinct emotional needs of men and women. For instance, water features could provide a calming environment for women while promoting relaxation and pleasure for men. Incorporating interactive elements like fountains or reflective pools may further amplify these therapeutic benefits.

The distinct emotional processing and aesthetic appreciation across genders also highlight the need to move beyond a one-size-fits-all approach in urban design. Everyday urban environments, such as public squares or pedestrian-friendly zones, benefit from designs that reduce visual clutter while ensuring aesthetic diversity. Coordinated floral arrangements or harmonized architectural styles can create visually pleasing spaces, particularly for women, while avoiding overly complex visual stimuli that might detract from overall well-being. However, these gender-specific responses may be most impactful in specialized therapeutic environments rather than in general urban contexts.

The use of FER technology in urban design research offers a valuable tool for assessing real-time emotional responses, providing actionable insights for creating inclusive and responsive urban environments. The application of Stress Reduction Theory (SRT) and Attention-Restoration Theory (ART) [71,72] in settings like rehabilitation centers, meditation zones, or urban parks can help optimize these spaces for emotional restoration. Future urban planning should adopt participatory design processes and leverage advanced tools such as AI-driven emotional analytics to refine designs, ensuring they are effective and inclusive for diverse user groups.

These findings establish the foundation for more nuanced urban design strategies, incorporating a variety of natural elements capable of evoking positive emotions and aesthetic pleasure. By addressing varying sensitivities and preferences, urban planning can contribute to the creation of more inclusive and livable cities that support the emotional well-being of all users.

### 4.4. Limitations and Future Outlook

This study introduces a pioneering approach to assessing gender-specific emotional responses to UGBS by integrating FER technology with self-report measures. Our experiments demonstrate the potential of FER in capturing nuanced emotional differences, which traditional self-report methods may overlook. While our study has laid the groundwork for understanding these dynamics, there are several avenues for future research and development. First, relying on GSV-based images or videos to represent UGBS may fail to fully capture the dynamic and immersive nature of real-world experiences. Future studies could use virtual reality (VR) simulations or complement lab-based experiments with field studies to provide a more holistic and realistic assessment of UGBS experiences [73,74]. Second, the current study serves as a proof of concept, conducted with a limited number of participants in the FER experiment due to the complexities involved in facial expression analysis (Table S1 summarizes the sample sizes used in related studies). Future work will expand this sample size to enhance the generalizability of our findings and provide a more comprehensive understanding of gender dynamics in emotional responses to UGBS. Third, we plan to extend our research to different urban environments and cultural contexts [70], leveraging the transferable nature of our methodological framework. This will allow us to explore the universality of our findings and tailor urban design strategies to meet the emotional needs of diverse populations globally. Fourth, relying solely on FER to assess emotional responses may not capture the full complexity of human emotions. We plan to incorporate additional physiological and socio-cultural variables into our model to develop a more individual-tailored, human-centric approach to urban green space design. For instance, we aim to include physiological measures such as heart rate variability, blood pressure, and skin conductance alongside facial expression analysis. This multimodal approach will enrich our data on each participant and possibly influence the model performance, providing a more comprehensive understanding of how different factors contribute to emotional responses in UGBS. Fifth, further investigation into other aspects of UGBS, such as safety, restorative potential, and social significance, is essential for understanding their effects on emotional perceptions [72,75,76]. These dimensions are crucial for a comprehensive understanding of UGBS effects on well-being and for informing inclusive urban design strategies. While our study provides valuable insights into gender differences in UGBS perception, there is a need for continued research to address these limitations and explore the broader implications of our findings.

## 5. Conclusions

The study utilized a dual-method approach, combining FER and self-reporting, to investigate gender differences in human perception and aesthetic preferences through panoramic video and static images. FER data tracked participants’ immediate and unconscious emotional reactions, providing insights into how urban elements influence human perception. Our findings reveal that changes in visual variables significantly impact perception across genders. Women typically exhibit more intense pleasant emotions in response to UGBS, while men show quicker reactions to visual changes. The study indicates that men are more likely to rate scenes higher when peak positive emotions are evoked, whereas women prefer scenes that induce higher arousal levels. The presence of grassy areas, for instance, can evoke delightful and relaxing emotions in women and arousal responses in men. Furthermore, blue spaces have a calming effect on both genders, with men experiencing increased relaxation as the presence of water grows. This comparative analysis deepened our understanding of gender-specific perceptions and demonstrated the advantages of FER technology in assessing real-time emotional responses compared to traditional self-reporting methods. The integration of FER into urban design research provides a robust tool for evaluating emotional impacts, enabling more nuanced and inclusive design strategies. Prioritizing gender-sensitive designs in UGBS by incorporating grassy areas, water features, and visually harmonious elements tailored to emotional needs, while leveraging participatory processes and advanced tools like FER, can create spaces that support emotional well-being and social cohesion. By addressing emotional dynamics and gender-specific insights, urban planners can design spaces that enhance mental health, foster inclusivity, and contribute to more livable and harmonious cities.

## Figures and Tables

**Table 1:** Socio-demographic and individual biographical information of participants.

Measures	Categories	Self-Report Experiment		Facial Recognition Experiment		Total		
		Male	Female	Male	Female	Male	Female	All
Amount		52 (40.6%)	56 (43.8%)	10 (7.8%)	10 (7.8%)	62 (48.4%)	66 (51.6%)	128 (100%)
Age	16–20	7 (5.5%)	11 (8.6%)	0 (0.0%)	1 (0.8%)	7 (5.5%)	12 (9.4%)	19 (14.8%)
	21–25	10 (7.8%)	4 (3.1%)	10 (7.8%)	8 (6.3%)	20 (15.6%)	12 (9.4%)	32 (25.0%)
	26–30	4 (3.1%)	8 (6.3%)	0 (0.0%)	1 (0.8%)	4 (3.1%)	9 (7.0%)	13 (10.2%)
	31–35	11 (8.6%)	15 (11.7%)	0 (0.0%)	0 (0.0%)	11 (8.6%)	15 (11.7%)	26 (20.3%)
	36–40	11 (8.6%)	15 (11.7%)	0 (0.0%)	0 (0.0%)	11 (8.6%)	15 (11.7%)	26 (20.3%)
	41–45	9 (7.9%)	3 (2.3%)	0 (0.0%)	0 (0.0%)	9 (7.9%)	3 (2.3%)	12 (9.4%)
	Mean	31.46	30.50	23.90	23.10	30.10	29.36	29.72
	S.D.	8.55	7.86	0.74	1.45	8.42	7.77	8.07
Race	Chinese	52 (40.6%)	56 (43.8%)	10 (7.8%)	10 (7.8%)	62 (48.4%)	66 (51.6%)	128 (100%)
Country living in before 15 years old	China	52 (40.6%)	56 (43.8%)	10 (7.8%)	10 (7.8%)	62 (48.4%)	66 (51.6%)	128 (100%)
Landscape/urban planning/architecture related field	Yes	11 (8.6%)	5 (3.9%)	0 (0.0%)	0 (0.0%)	11 (8.6%)	5 (3.9%)	16 (12.5%)
	No	41 (32.0%)	49 (38.3%)	10 (7.8%)	10 (7.8%)	51 (39.8%)	61 (47.7%)	112 (87.5%)
Have been to UK	Yes	4 (3.1%)	3 (2.3%)	0 (0.0%)	0 (0.0%)	4 (3.1%)	3 (2.3%)	7 (5.5%)
	No	48 (37.5%)	53 (41.4%)	10 (7.8%)	10 (7.8%)	58 (45.3%)	63 (49.2%)	121 (94.5%)
Have been to Japan	Yes	3 (2.3%)	0 (0.0%)	0 (0.0%)	0 (0.0%)	3 (2.3%)	0 (0.0%)	3 (2.3%)
	No	49 (38.3%)	56 (43.8%)	10 (7.8%)	10 (7.8%)	59 (46.1%)	66 (51.6%)	125 (97.7%)

**Table 2:** Descriptive statistics of perception measures by gender across nine scenes.

Clip	Measures	Men			Women		
		N	Mean	SD	N	Mean	SD
C1	Valence_FER	240	0.03	0.18	240	0.01	0.23
	Arousal_FER	240	0.32	0.11	240	0.32	0.08
	Preference_FER	10	0.21	0.15	10	0.27	0.11
	Valence_SAM	52	0.14	0.41	56	0.16	0.47
	Arousal_SAM	52	−0.01	0.48	56	−0.06	0.49
	Preference_SAM	52	0.19	0.45	56	0.11	0.45
C5	Valence_FER	240	−0.01	0.18	240	0.00	0.25
	Arousal_FER	240	0.33	0.14	240	0.33	0.08
	Preference_FER	10	0.15	0.14	10	0.29	0.10
	Valence_SAM	52	0.20	0.45	56	0.14	0.48
	Arousal_SAM	52	−0.01	0.45	56	−0.05	0.45
	Preference_SAM	52	0.12	0.45	56	0.16	0.46
C7	Valence_FER	240	0.05	0.15	240	−0.01	0.16
	Arousal_FER	240	0.30	0.12	240	0.29	0.06
	Preference_FER	10	0.06	0.16	10	0.13	0.13
	Valence_SAM	52	0.12	0.47	56	0.11	0.44
	Arousal_SAM	52	0.04	0.45	56	−0.07	0.42
	Preference_SAM	52	0.13	0.44	56	0.05	0.42
C9	Valence_FER	240	0.05	0.15	240	0.03	0.23
	Arousal_FER	240	0.31	0.12	240	0.29	0.08
	Preference_FER	10	0.11	0.10	10	0.17	0.12
	Valence_SAM	52	0.21	0.48	56	0.23	0.46
	Arousal_SAM	52	0.00	0.47	56	−0.02	0.45
	Preference_SAM	52	0.17	0.47	56	0.21	0.42
C11	Valence_FER	240	0.04	0.14	240	0.00	0.19
	Arousal_FER	240	0.30	0.10	240	0.31	0.07
	Preference_FER	10	0.18	0.10	10	0.28	0.06
	Valence_SAM	52	0.21	0.39	56	0.23	0.47
	Arousal_SAM	52	0.09	0.43	56	0.05	0.48
	Preference_SAM	52	0.24	0.42	56	0.23	0.44
C13	Valence_FER	240	0.02	0.17	240	0.04	0.20
	Arousal_FER	240	0.32	0.10	240	0.30	0.09
	Preference_FER	10	0.08	0.11	10	0.15	0.08
	Valence_SAM	52	0.25	0.40	56	0.20	0.48
	Arousal_SAM	52	0.04	0.47	56	0.02	0.46
	Preference_SAM	52	0.23	0.44	56	0.19	0.43
C15	Valence_FER	240	0.04	0.15	240	0.03	0.23
	Arousal_FER	240	0.30	0.10	240	0.29	0.08
	Preference_FER	10	0.16	0.13	10	0.27	0.13
	Valence_SAM	52	0.21	0.40	56	0.15	0.42
	Valence_SAM	52	0.21	0.40	56	0.15	0.42
	Arousal_SAM	52	0.05	0.44	56	0.02	0.45
	Preference_SAM	52	0.23	0.39	56	0.18	0.45
C17	Valence_FER	240	0.06	0.20	240	0.02	0.18
	Arousal_FER	240	0.33	0.11	240	0.27	0.08
	Preference_FER	10	0.03	0.16	10	0.11	0.10
	Valence_SAM	52	0.23	0.40	56	0.17	0.47
	Arousal_SAM	52	0.11	0.43	56	0.04	0.46
	Preference_SAM	52	0.25	0.45	56	0.19	0.43
C19	Valence_FER	240	0.10	0.12	240	0.03	0.22
	Arousal_FER	240	0.30	0.08	240	0.30	0.06
	Preference_FER	10	0.22	0.10	10	0.20	0.14
	Valence_SAM	52	0.23	0.38	56	0.10	0.44
	Arousal_SAM	52	0.05	0.42	56	0.00	0.45
	Preference_SAM	52	0.18	0.45	56	0.13	0.47

Notes: Valence_FER: Valence perception data obtained from the FER method. Arousal_FER: Arousal perception data obtained from the FER method. Preference_FER: Aesthetic preference ratings obtained in the facial expression experiment. Valence_SAM: Valence perception data obtained from the SAM scale. Arousal_SAM: Arousal perception data obtained from the SAM scale. Preference_FER: Aesthetic preference ratings obtained in the self-reported emotion experiment.

**Table 3:** Model 1: Gender differences in backward stepwise regression on the relationship between basic visual variables and FER emotional data.

**Visual** **Variables**	**Male**			
	**Valence_FER** **Adj. R^2^ = 0.076**		**Arousal_FER** **Adj. R^2^ = 0.034**	
	***t*-Value**	**95%CI**	***t*-Value**	**95%CI**
GVI			2.923 **	[0.010, 0.051]
Sky	4.336 **	[0.073, 0.193]		
Building				
Constant	0.460	[−1.423, 2.288]	43.759 **	[28.050, 30.696]
**Visual** **Variables**	**Female**			
	**Valence_FER** **Adj. R^2^ = 0.135**		**Arousal_FER** **Adj. R^2^ = 0.000**	
	***t*-Value**	**95%CI**	***t*-Value**	**95%CI**
GVI	4.737 **	[0.134, 0.324]		
Sky	5.961 **	[0.207, 0.411]		
Building	3.700 **	[0.104, 0.342]		
Constant	−4.902 **	[−31.952, −13.624]	196.744 **	[29.708, 30.310]

Note: ** ≤ 0.01. Model 1 included GVI, Sky, and Building as independent variables.

**Table 4:** Model 2: Gender differences in backward stepwise regression on the relationship between finer visual variables and FER emotional data.

**Visual** **Variables**	**Male**			
	**Valence_FER** **Adj. R^2^ = 0.164**		**Arousal_FER** **Adj. R^2^ = 0.045**	
	***t*-Value**	**95%CI**	***t*-Value**	**95%CI**
VPI			3.322 **	[0.013, 0.049]
Water	4.834 **	[0.080, 0.189]		
Sky	4.633 **	[0.078, 0.193]		
Building				
Constant	−0.227	[−1.990, 1.579]	52.377 **	[28.371, 30.590]
**Visual** **Variables**	**Female**			
	**Valence_FER** **Adj. R^2^ = 0.131**		**Arousal_FER** **Adj. R^2^ = 0.178**	
	***t*-Value**	**95%CI**	***t*-Value**	**95%CI**
VPI			6.996 **	[0.072, 0.128]
Water	4.387 **	[0.125, 0.329]		
Sky	5.915 **	[0.206, 0.413]	4.541 **	[0.055, 0.138]
Building	3.688 **	[0.104, 0.342]	4.035 **	[0.047, 0.137]
Constant	−4.879 **	[−32.069, −13.612]	15.209 **	[18.232, 23.662]

Note: ** ≤ 0.01. Model 2 included VPI, Water, Sky, and Building as independent variables.

**Table 5:** Model 3: Gender differences in backward stepwise regression on the relationship between the most detailed visual variables and FER emotional data.

**Visual** **Variables**	**Male**			
	**Valence_FER** **Adj. R^2^ = 0.258**		**Arousal_FER** **Adj. R^2^ = 0.108**	
	***t*-Value**	**95%CI**	***t*-Value**	**95%CI**
Tree	−6.101 **	[−0.237, −0.121]	−1.865 *	[−0.088, 0.002]
Grass				
Shrub	−4.152 **	[−0.080, −0.028]	−4.313 **	[−0.060, −0.022]
Water	4.097 **		−3.073 **	[−0.078, −0.017]
Sky		[0.059, 0.169]	−3.089 **	[−0.127, −0.028]
Building	−2.377 *	[−0.195, −0.018]	−3.527 **	[−0.144, −0.041]
Constant	10.485 **	[6.968, 10.195]	27.773 **	[33.079, 38.133]
**Visual** **Variables**	**Female**			
	**Valence_FER** **Adj. R^2^ = 0.175**		**Arousal_FER** **Adj. R^2^ = 0.245**	
	***t*-Value**	**95%CI**	***t*-Value**	**95%CI**
Tree			7.131 **	[0.128, 0.226]
Grass	5.813 **	[0.049, 0.099]	6.480 **	[0.062, 0.117]
Shrub			8.207 **	[0.096, 0.157]
Water	1.808	[−0.004, 0.087]		
Sky	1.851	[−0.003, 0.095]	6.525 **	[0.121, 0.226]
Building			5.487 **	[0.092, 0.195]
Constant	−2.318 *	[−3.177, −0.257]	10.314 **	[13.586, 20.007]

Note: * ≤ 0.05, ** ≤ 0.01. Model 3 included Tree, Grass, Shrub, Water, Sky and Building as independent variables.

**Table 6:** Two-tailed Pearson correlations between aesthetic preference and two SAM data for both genders.

Aesthetic Preference	Pearson’s r, *p* Value			
	Male		Female	
Valence_SAM	0.801 **	0.000	0.702 **	0.000
Arousal_SAM	0.487 **	0.000	0.433 **	0.000

Note: ** ≤ 0.01.

**Table 7:** Two-tailed Pearson correlations between aesthetic preference and six calculated FER emotional perception data for both genders.

Aesthetic Preference	Pearson’s r, *p* Value			
	Male		Female	
Valence_FER_max	0.249 *	0.018	0.205	0.053
Valence_FER_ave	0.094	0.292	0.205	0.053
Valence_FER_min	0.112	0.292	0.205	0.053
Arousal_FER_max	−0.037	0.732	0.205	0.053
Arousal_FER_ave	0.010	0.926	0.298 **	0.004
Arousal_FER_min	0.017	0.873	0.158	0.137

Note: * ≤ 0.05, ** ≤ 0.01. Valence_FER_max: The maximum FER valence data of each video clip. Valence_FER_ave: The average FER valence data of each video clip. Valence_FER_min: The minimum FER valence data of each video clip. Arousal_FER_max: The maximum FER arousal data of each video clip. Arousal_FER_ave: The average FER arousal data of each video clip. Arousal_FER_min: The minimum FER arousal data of each video clip.

## Data Availability

All shared data will be processed to exclude any content that involves participant privacy. For data requests, please contact zhang_xuan@zju.edu.cn.

## Supplementary Materials

The following supporting information can be downloaded at: https://www.mdpi.com/article/10.3390/s25030748/s1, Questionnaire S1: Personal Background Information; Questionnaire S2: Aesthetic Preference; Questionnaire S3: SAM Scale; Figure S1: Based on hashtag rankings, the study (A) selected ten cultural heritage sites and (B) created primary stimulation through panoramas taken from specific observation points at each site. The symbol “CX” in the upper left corner of each panorama denotes the display order for the related video clips; Figure S2: Screenshots of the primary stimulation panoramic video clips; Figure S3: FER model architecture and workflow. The images are from the Aff-wild2 dataset, which is publicly available and designed for facial expression recognition research; Table S1: Statistics on the number of volunteers in relevant studies. References [29,77,78,79,80,81] are cited in the supplementary materials.
