# Supplementary material for: Real-Time Sensor-Based and Self-Reported Emotional Perceptions of Urban Green-Blue Spaces: Exploring Gender Differences with FER and SAM
Source: Sensors (Basel). 2025 Jan 26;25(3):748. doi: 10.3390/s25030748 (PMC11820289; doi:10.3390/s25030748)
Supplement: Supplementary file 1 [file sensors-25-00748-s001.zip › sensors-3402618-supplementary/Questionnaire S1.pdf]

Questionnaire S1. Personal Background Information

1. Your Name:

____

2. Your Gender:

○Male ○Female

3. Your Age Group:

○15 years and below ○16-20 years ○21-25 years ○26-30 years

○31-35 years ○36-40 years ○41-45 years ○46-50 years

○51-55 years ○56-60 years ○61-65 years ○66-70 years

○Above 70 years

4. Your Education Level:

○Junior High School and below

○High School/Technical Secondary School

○Associate Degree

○Bachelor's Degree

○Postgraduate and above

5. Do you have a background in architecture, urban planning, landscape architecture, or related fields?

○ Yes

○ No

6. Your Marital Status:

○Single

○Married

○Divorced

○Widowed

7. Have you been to the UK?

○Yes

○No

8. Please rate your level of knowledge in the following areas on a scale from 1 to 10, where 1 indicates "Not at all knowledgeable" and 10 indicates "Extremely knowledgeable":

	1	2	3	4	5	6	7	8	9	10
Perception of UK Garden Culture	○	○	○	○	○	○	○	○	○	○
Perception of UK Gardening Style	○	○	○	○	○	○	○	○	○	○
Perception of UK Horticulture	○	○	○	○	○	○	○	○	○	○
Perception of UK Architecture Style	○	○	○	○	○	○	○	○	○	○
Overall Country Impression of the UK	○	○	○	○	○	○	○	○	○	○

9. Have you been to Japan?

○Yes

○No

8. Please rate your level of knowledge in the following areas on a scale from 1 to 10, where 1 indicates "Not at all knowledgeable" and 10 indicates "Extremely knowledgeable":

	1	2	3	4	5	6	7	8	9	10
Perception of Japanese Garden Culture	○	○	○	○	○	○	○	○	○	○
Perception of Japanese Gardening Style	○	○	○	○	○	○	○	○	○	○
Perception of Japanese Horticulture	○	○	○	○	○	○	○	○	○	○
Perception of Japanese Architecture Style	○	○	○	○	○	○	○	○	○	○
Overall Country Impression of Japan	○	○	○	○	○	○	○	○	○	○
