# Supplementary material for: Real-Time Sensor-Based and Self-Reported Emotional Perceptions of Urban Green-Blue Spaces: Exploring Gender Differences with FER and SAM
Source: Sensors (Basel). 2025 Jan 26;25(3):748. doi: 10.3390/s25030748 (PMC11820289; doi:10.3390/s25030748)
Supplement: Supplementary file 1 [file sensors-25-00748-s001.zip › sensors-3402618-supplementary/Questionnaire S2.pdf]

Questionnaire S2. Aesthetic Preference

1. Please rate your level of preference for this landscape on a scale of 1 to 10, where 1 indicates "Strongly Dislike" and 10 indicates "Strongly Like".

	1	2	3	4	5	6	7	8	9	10
Aesthetic Preference	○	○	○	○	○	○	○	○	○	○
