# Supplementary material for: Real-Time Sensor-Based and Self-Reported Emotional Perceptions of Urban Green-Blue Spaces: Exploring Gender Differences with FER and SAM
Source: Sensors (Basel). 2025 Jan 26;25(3):748. doi: 10.3390/s25030748 (PMC11820289; doi:10.3390/s25030748)

**Questionnaire S3.** SAM Scale

1. Please select the degree of pleasure you feel after watching the panoramic video of this scene. The numbers 1, 3, 5, 7 and 9 represent frown (a frowning figure), unhappy, neutral, smiling, and happy ( a smiling figure). Numbers 2, 4, 6, 8 represent subtler gradations between these levels.

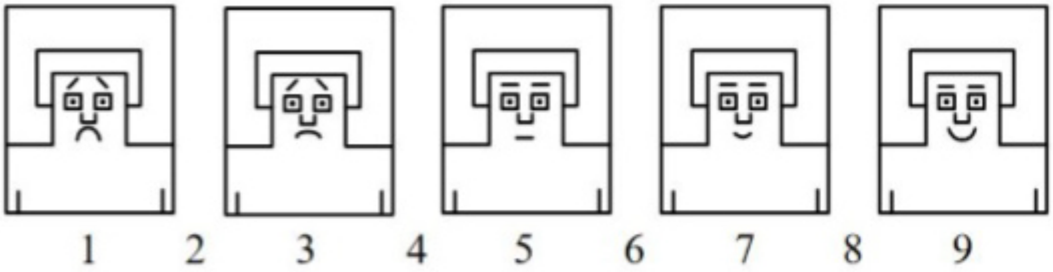


2. Please rate the level of arousal you feel after watching the panoramic video of this scene. The numbers 1, 3, 5, 7 and 9 indicate sleepy (eyes closed), drowsy, neutral, excited, and stimulated (eyes wide open). Numbers 2, 4, 6, 8 indicate subtler gradations between these levels.

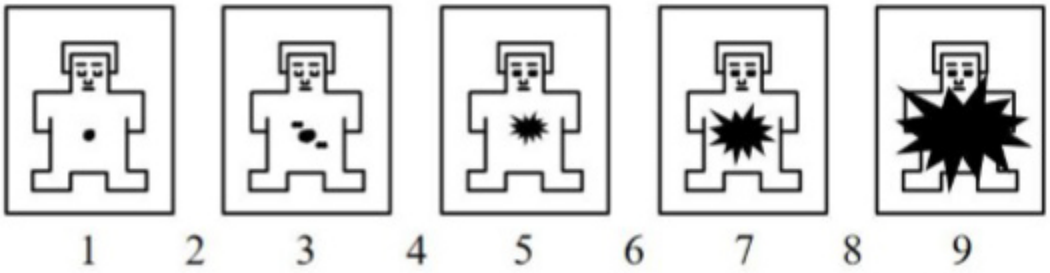

Supplement: Supplementary file 1 [file sensors-25-00748-s001.zip › sensors-3402618-supplementary/Questionnaire S3.pdf]
