# Supplementary material for: Real-Time Sensor-Based and Self-Reported Emotional Perceptions of Urban Green-Blue Spaces: Exploring Gender Differences with FER and SAM
Source: Sensors (Basel). 2025 Jan 26;25(3):748. doi: 10.3390/s25030748 (PMC11820289; doi:10.3390/s25030748)
Supplement: Supplementary file 1 [file sensors-25-00748-s001.zip › sensors-3402618-supplementary/Table S1.pdf]

Supplementary Materials

Number of volunteers in relevant studies

Table S1. Statistics on the number of volunteers in relevant studies.

Reference	Research contents	Composition of volunteers
(Liu et al., 2023)	Emotional perception of human comfort on the sidewalks	**15** college students
(Wang et al., 2022)	Six emotional perceptions of beautiful, boring, depressing, lively, wealthy, and safety	**30** volunteers
(Qiu et al., 2021)	Four emotional perceptions of enclosure, human scale, complexity, and imageability	**23** volunteers
(Ma et al., 2021)	Five emotional perceptions of greenness, openness, enclosure, walkability, and imageability	**5** volunteers
(Ye et al., 2019)	The street view was greenery enough or not yet	**10** urban design experts
(Yao et al., 2019)	Six emotional perceptions of beautiful, boring, depressing, lively, wealthy, and safety	**20** college students and staff
**This study**	**Women's and men's two emotional perceptions of valence and arousal towards UGBS**	**108 citizens for self-reported SAM**<br>**20 college students for FER experiment**

References

Liu, P., Zhao, T., Luo, J., Lei, B., Frei, M., Miller, C., Biljecki, F., 2023. Towards Human-centric Digital Twins: Leveraging Computer Vision and Graph Models to Predict Outdoor Comfort. Sustain. Cities Soc. 93, 104480. https://doi.org/10.1016/j.scs.2023.104480

Ma, X., Ma, C., Wu, C., Xi, Y., Yang, R., Peng, N., Zhang, C., Ren, F., 2021. Measuring human perceptions of streetscapes to better inform urban renewal: A perspective of scene semantic parsing. Cities 110, 103086. https://doi.org/10.1016/j.cities.2020.103086

Qiu, W., Li, W., Liu, X., Huang, X., 2021. Subjectively Measured Streetscape Perceptions to Inform Urban Design Strategies for Shanghai. ISPRS Int. J. Geo-Inf. 10, 493. https://doi.org/10.3390/ijgi10080493

Wang, L., Han, X., He, J., Jung, T., 2022. Measuring residents' perceptions of city streets to inform better street planning through deep learning and space syntax. ISPRS J. Photogramm. Remote Sens. 190, 215–230. https://doi.org/10.1016/j.isprsjprs.2022.06.011

Yao, Y., Liang, Z., Yuan, Z., Liu, P., Bie, Y., Zhang, J., Wang, R., Wang, J., Guan, Q., 2019. A human-machine adversarial scoring framework for urban perception assessment using street-view images. Int. J. Geogr. Inf. Sci. 33, 2363–2384. https://doi.org/10.1080/13658816.2019.1643024

Ye, Y., Richards, D., Lu, Y., Song, X., Zhuang, Y., Zeng, W., Zhong, T., 2019. Measuring daily accessed street greenery: A human-scale approach for informing better urban planning practices. Landsc. Urban Plan. 191, 103434. https://doi.org/10.1016/j.landurbplan.2018.08.028
