# Supplementary figures and images for: Real-Time Sensor-Based and Self-Reported Emotional Perceptions of Urban Green-Blue Spaces: Exploring Gender Differences with FER and SAM
Source: Sensors (Basel). 2025 Jan 26;25(3):748. doi: 10.3390/s25030748 (PMC11820289; doi:10.3390/s25030748)

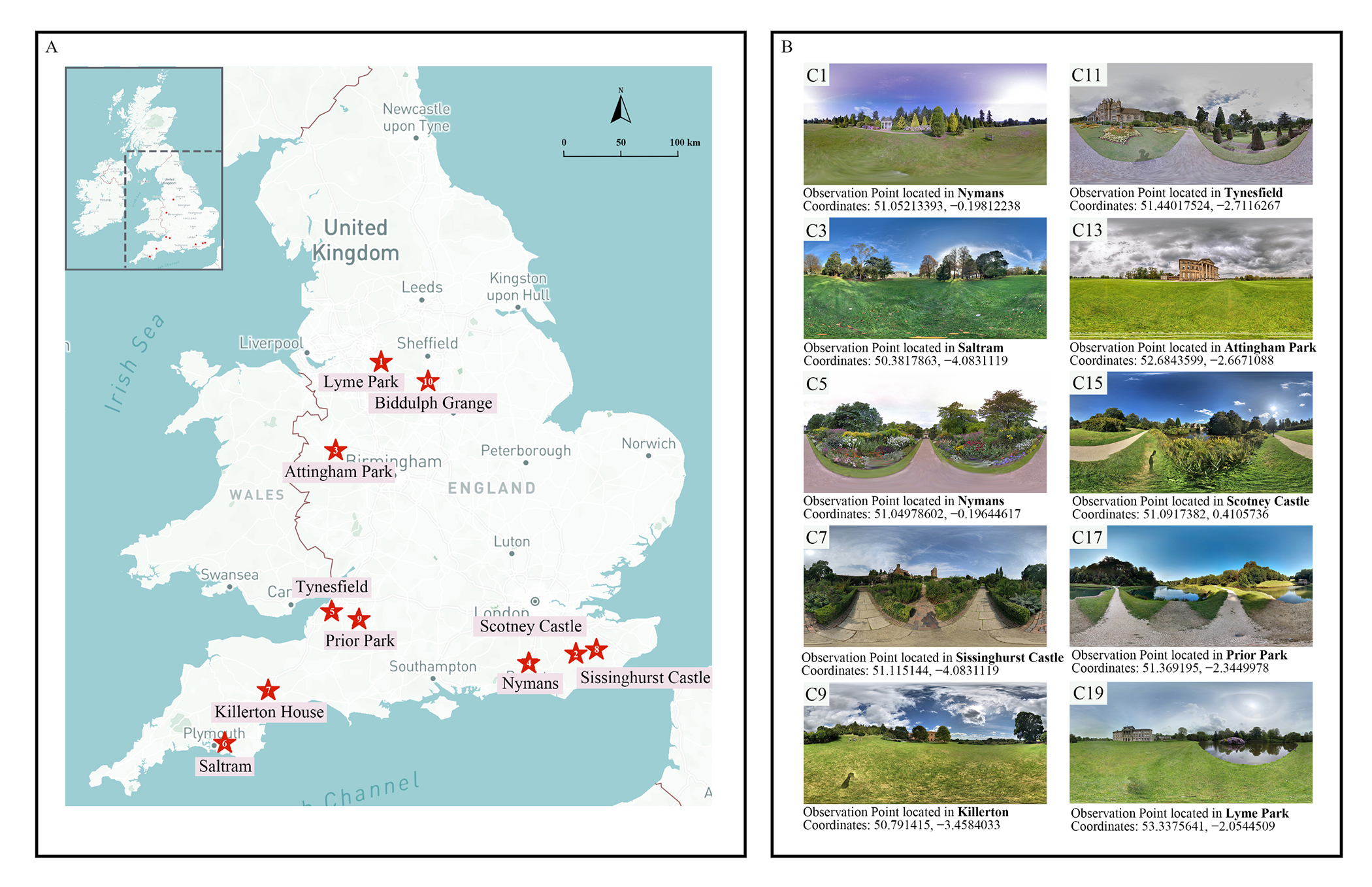

Supplement: Supplementary file 1 [file sensors-25-00748-s001.zip › sensors-3402618-supplementary/Figure S1.jpg]

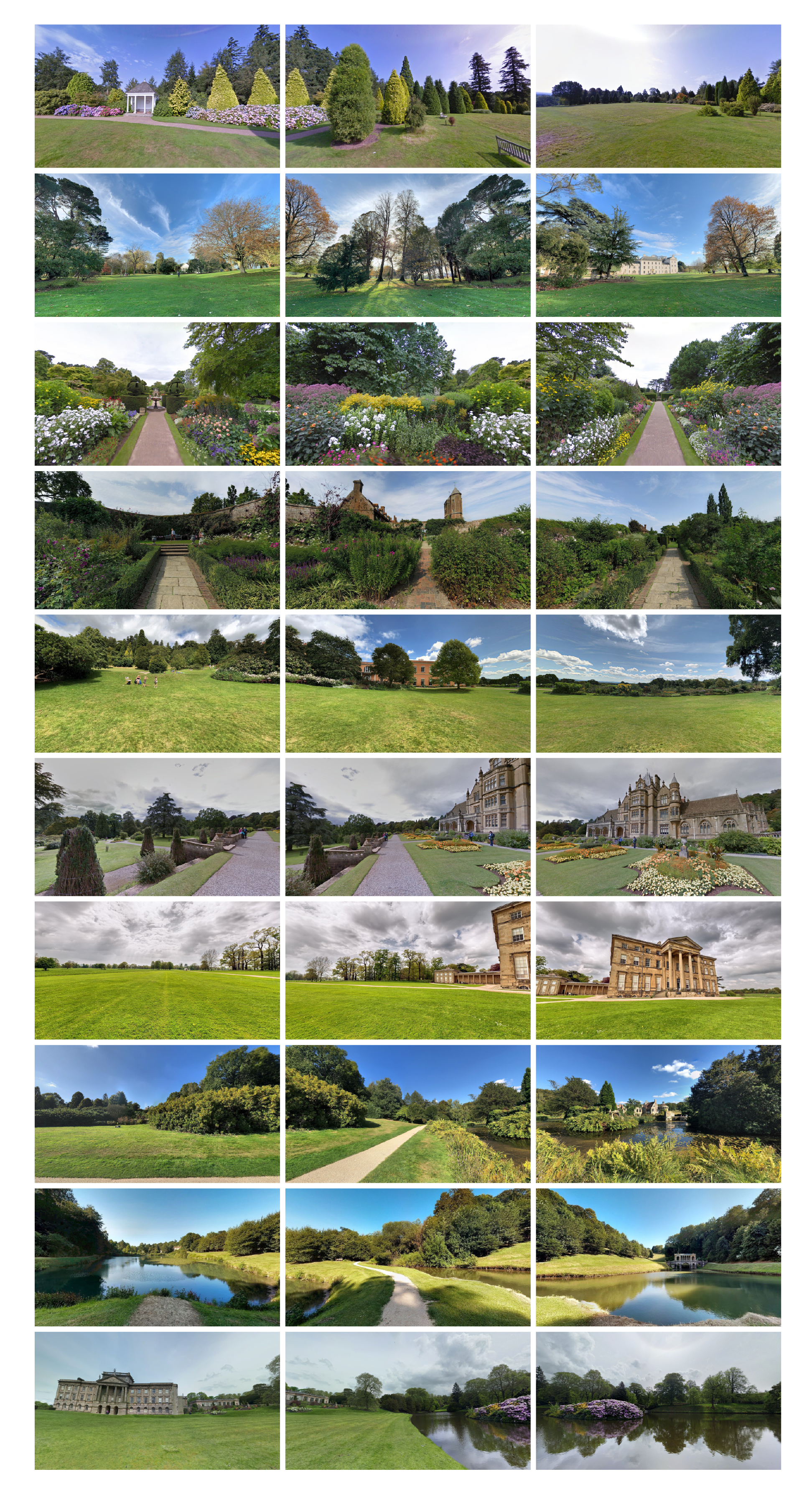

Supplement: Supplementary file 1 [file sensors-25-00748-s001.zip › sensors-3402618-supplementary/Figure S2.png]

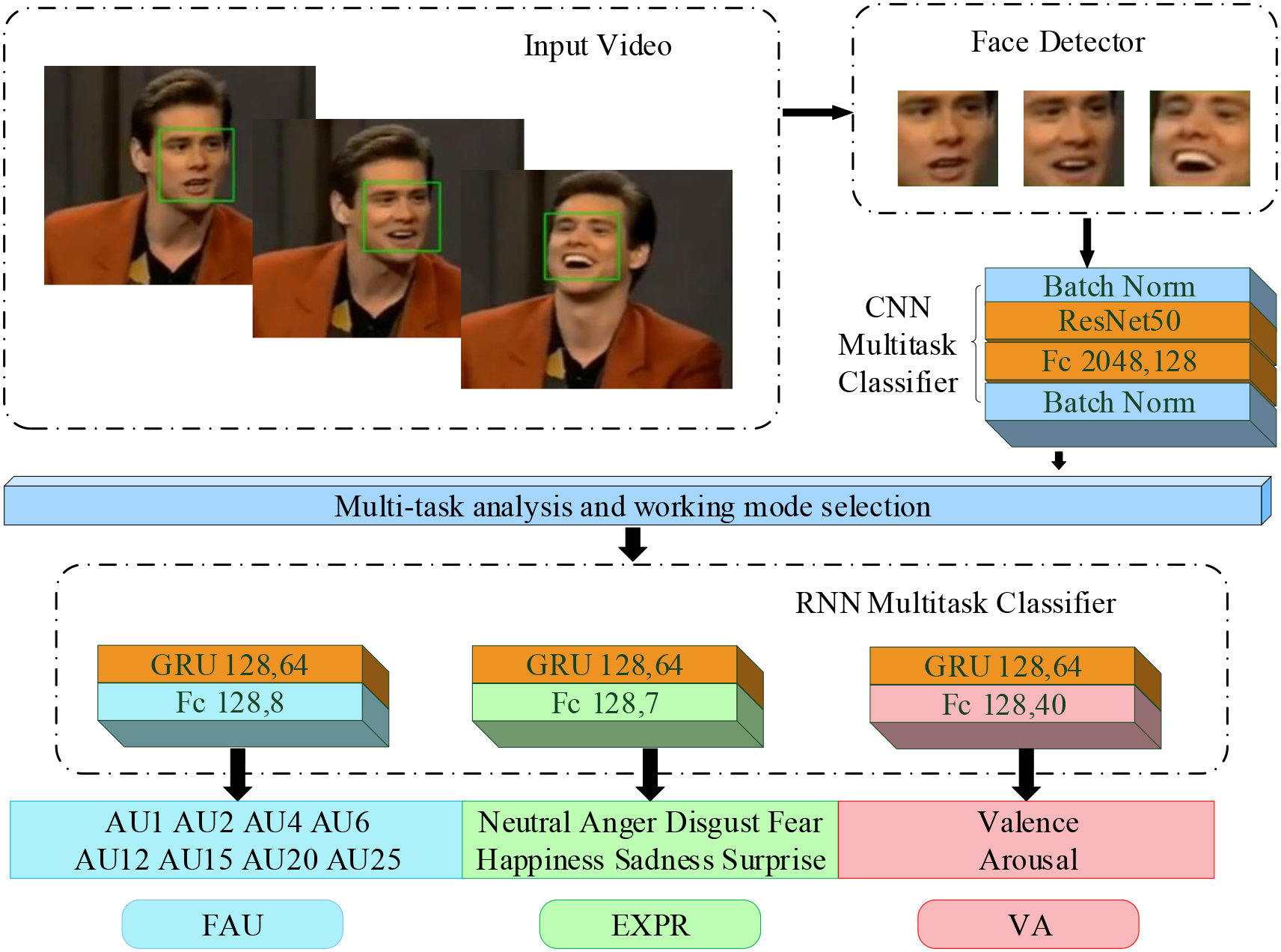

Supplement: Supplementary file 1 [file sensors-25-00748-s001.zip › sensors-3402618-supplementary/Figure S3.jpg]
